# Adverse effects of homeopathy: a systematic review of published case reports and case series – response by Posadzki and Ernst

**DOI:** 10.1111/ijcp.12140

**Published:** 2013-04

**Authors:** P Posadzki, E Ernst

**Affiliations:** Complementary Medicine, University of ExeterExeter, UK

## Abstract

**Linked Comment:** Jackson. *Int J Clin Pract* 2013; 67: 385.

**Linked Comment:** Walach *et al*. *Int J Clin Pract* 2013; 67: 385–6.

**Linked Comment:** Posadzki and Ernst. *Int J Clin Pract* 2013; 67: 386–7.

**Linked Comment:** Grimes. *Int J Clin Pract* 2013; 67: 387.

**Linked Comment:** Tournier *et al*. *Int J Clin Pract* 2013; 67: 388–9.

We would like to respond to the comments by Tournier et al. (1) as follows:

- The issues regarding the case report of Geukens, were addressed in our previous response (2).
- Regarding the articles by Barquero-Romero (2004), Lim (2011), Luder (2000) and Prasad (2006) we see no good reason for the notion that the four deaths following ingestion of homeopathic remedies could have been prevented if a ‘competent qualified homeopath’ had acted more ‘responsibly’. In fact, we very broadly covered the issues of some homeopaths' professional irresponsibility and its consequences in the discussion section.
- Tournier et al., seem to confuse the report by Zuzak et al. (2010) with the one by Von Mach et al. (2006). The former included nine cases of intoxications following the ingestion of homeopathic remedies, whereas the latter reported the figure of 1070 cases. In the case series by Zuzak, the 2143 cases were omitted because they referred not to therapeutic but to accidental intake of homeopathic remedies. Perhaps we should have included those cases too. Then the total number of patients who experienced AEs of homeopathy would have amounted to 3293!
- We provided clear definitions of homeopathy in the introduction to our review.
- Sasseville (1995) provided full details of the composition of the ointments along with the level of dilution, e.g. *Rhus tox* (2CH). Tincture of aconite presented in the case by Guha et al. (1999) is technically speaking, a homeopathic remedy. Tournier et al. know of course that the method of preparation of *Aconitum napellus* varies in different pharmacopoeias and therefore safety issues arise when these differences are neglected (2, 3).
- In our view, the data extraction of the CR by Bernez et al. (2008) and its interpretations were correct. We regret, however, that the translation of the Danish text has led to confusion.

In view of these arguments, we reject the accusation of Tournier et al. that our results (4) are unreliable. We strongly believe that the conclusions of our review were justified.
